# Expression of Migration-Related Genes in Human Colorectal Cancer and Activity of a Disintegrin and Metalloproteinase 17 

**DOI:** 10.1155/2016/8208904

**Published:** 2016-03-27

**Authors:** Katarzyna Walkiewicz, Paweł Kozieł, Martyna Bednarczyk, Adam Błażelonis, Urszula Mazurek, Małgorzata Muc-Wierzgoń

**Affiliations:** ^1^Department of Internal Medicine, Faculty of Public Health in Bytom, Medical University of Silesia, 40-055 Katowice, Poland; ^2^Department of Molecular Biology, Faculty of Pharmacy in Sosnowiec, Medical University of Silesia, 40-055 Katowice, Poland

## Abstract

*Introduction.* The ability to form metastases which depends on the mechanisms of cell migration is an important element of the progression of cancer. In the present study we analyzed the genes involved in the regulation of migration in colon cancer cells.* Materials and Methods.* A total of 20 pairs of surgically removed tumoral and healthy (marginal) tissues samples from colorectal cancer patients at clinical stages I-II and III-IV were analyzed. The isolation of RNA from CRC and normal tissues and its subsequent molecular analysis were performed according to manufacturer's instructions. Microarray data analysis was performed using the GeneSpring 11.5 platform and Significance Analysis of Microarrays (SAM). In SAM analysis to identify significantly differentially expressed genes score and *q*-value parameters were used.* Results.* The largest increase in expression of genes was shown by MMP9, ADAM17, EphA2, and TIMP.* Conclusions.* Presented genes, especially ADAM17, MMP9, EphA2, TIMP1, ICAM 11, and CD4, may be used as prognostic markers of advanced stages of colorectal cancer, contributing to the development of new lines of therapy focused on reducing metastasis of the primary tumor.

## 1. Introduction

The formation of metastasis is complex and dependent on, inter alia, proteolytic activity of tumor cells, their ability of migration, proliferative activity, and the ability of neovascularization [1]. The steps of this process described the “cascade of metastasis” as follows:

1. Detachment of cells from the primary tumor.
2. Cell migration and penetration of blood vessels/lymph.
3. Cell survival in the circulation.
4. Leaving the cells of blood vessels and organs settlement.
5. The growth of tumor cells [2].

Cancer cells are able to spread in the body using two mechanisms, invasion and the metastasis, meaning the ability of tumor cells to penetrate the walls of blood and lymphatic vessels and to be transported to other often distant tissues and create secondary tumors. Migrant cell is characterized by a loss of intercellular connections, resulting in the absence or low expression of E-cadherin [3, 4]. The loss of the interaction is caused by a change of gene expression of adhesion molecules and proteolytic enzymes and mutations in genes regulating the migration and cell cycle [3]. Tumor cells exceed the basal membrane due to the ability to secrete serine and cysteine proteases, matrix metalloproteinases (MMPs), and plasminogen activators [4]. MMPs are called collagenoses. Together with three other families of proteins, astacynases, adamalizynes, and serralizynes form a superfamily, endoproteinases (called metzyncynases), which participate in the processes of reception of signals from the environment, activation, proliferation, differentiation, and apoptosis [5–9]. Metalloproteinase activity is controlled at many levels and includes the transcription and events of posttranslational inhibition of the metalloproteinase inhibitors stabilizing mRNA, secretion and activation proenzymes, and proteolysis [5]. The ability to inhibit the proteolytic activity of matrix metalloproteinases has also TIMPs (tissue inhibitors of metalloproteinases) [10].

A disintegrin and metalloproteinase-17 (ADAM17, also named as tumor necrosis factor-alpha-converting enzyme, TACE) is expressed in most tissues and is upregulated during inflammation, tumor growth, and angiogenesis [11]. ADAM17 has been initially identified as the main sheddase responsible for releasing the soluble form of tumor necrosis factor (TNF) from the plasma membrane and is known to shed a variety of growth factors, receptors, and adhesion molecules, such as epidermal growth factor receptor (EGFR) ligands, p75 TNF receptor, interleukin-1 receptor type II, p55 TNF receptor, transforming growth factor-alpha, L-selectin, growth hormone receptor, MUC1, and the amyloid precursor protein [12]. In fact, as many as 76 proteins have been shown to be substrates for ADAM17 shedding activity, thus regulating responses to tissue injury, inflammation, and carcinogenesis [13]. It is indispensable regulator of cellular events from proliferation to migration [14]. The high expression of ADAM17 genes is poor prognostic factor in various cancer types and correlates with tumor progression (e.g., breast, prostate, gastric, colorectal, hepatocellular, and ovarian cancer) [15].

Colorectal cancer is the third most common cancer. Its development involves many steps of genetic changes such as inactivation of tumor suppressor genes and activation of oncogenes, often associated with the progression of premalignant adenomas to invasive adenocarcinoma [16].

The research which focused on finding sensitive and specific methods to assess diagnostic and prognostic colorectal cancer estimated that more purposeful exploration of the individual markers of carcinogenesis is to determine the genetic profile of colorectal cancer.

Aim of this study is to predict metastasis-related genes differentiating colorectal cancer tissue from healthy tissue depending on the clinical stage of the disease and identify the potential role of ADAM17 in this process.

## 2. Materials and Methods

### 2.1. Tissue Sampling

A total of 20 pairs of surgically removed tumoral and healthy (marginal) tissues samples from colorectal cancer patients at clinical stages I-II and III-IV were collected. Healthy control tissue specimens (marked C) were obtained from an area of 10 mm outside of the histologically negative margin. The tumor specimens were divided into two groups according to the 7th edition of the AJCC/UICC staging system of CRC: cases of colorectal cancer (CRC) were divided into “low stage of cancer” (I–IIC) and “high stage of cancer” (IIIA–IVB).

Samples were placed in RNAlater reagents and stored at –80°C.

The study protocol was approved by the Bioethical Committee of the Medical University of Silesia (KNW/0022/KB1/42/14), and informed consent was obtained from all patients.

### 2.2. Method

The isolation of RNA from CRC and normal tissues (Invitrogen Life Technologies, USA) and its subsequent molecular analysis (Gene Expression Analysis Technical Manual procedures) were performed according to manufacturer's instructions. Microarray data analysis was performed using the GeneSpring 11.5 platform (Agilent Technologies UK Limited, South Queensferry, United Kingdom) and Significance Analysis of Microarrays (SAM). In SAM analysis to identify significantly differentially expressed genes score and *q*-value parameters were used. The array data were shown by volcano plots, which are one of the best mathematical representations available to compare the expressions of multiple genes from two samples. The results were analyzed with particular consideration to *P* value and fold change (FC) parameters. The parameter of *P* value indicates the percentage probability of accidental occurrence of the observed differences in fluorescence signal. Its value should be lower than 0.05. The fold change (FC) determines the degree of variation of studied transcriptomes fluorescence against control subjects. Its value should be higher than 1.1.

The expression patterns of the controls were compared to differentially expressed genes identified in LSC and HSC specimens. Differences in gene expression were evaluated using univariate analysis of variance, ANOVA, and Tukey's post hoc multiple comparisons test, both with Benjamini-Hochberg correction.

## 3. Results

The results were repeated two times in each subgroup. The comparison analysis of the transcriptomes that were identified by microarray (Affymetrix) was conducted. Out of the collection of 22 283 ID mRNA that were located on the HG-U133A microarray, 909 ID mRNA were selected using the NetAffx*™* database. Using the Affymetrix scientific database and available literature data, 497 genes for further analysis of transcriptomes were typed.

### 3.1. Comparing Transcriptomes Derived from Colorectal Cancer Specimens with Healthy Tissues

**Figure 1:**
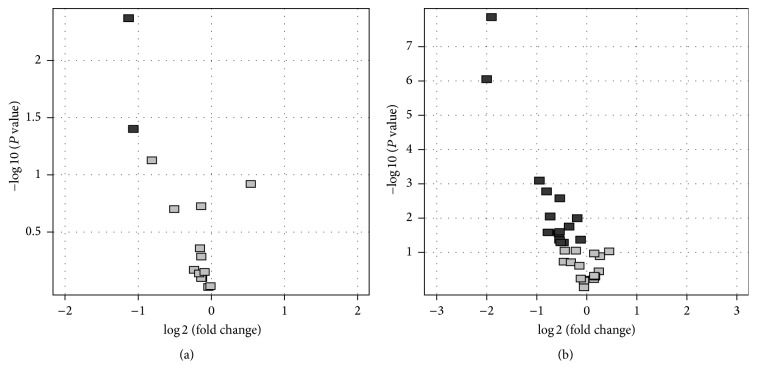
Genes differentiating: (a) LSC versus C and (b) HCS versus C; grey color, all genes with *P* < 0.05; black color, genes with FC > 1.1 and *P* < 0.05.

Differentiation of genes of colorectal cancer tissues versus control was presented graphically on Figure Volcano (Figure 1) and in Tables 1 and 2. The figure illustrates the distribution of the fluorescence signal depending on the changes in gene expression between colorectal cancer and control cells on the basis of negative values of log⁡10 (*P* value) versus log⁡2 (fold change).

### 3.2. Designation of Genes Differentiating Transcriptomes of LSC Specimens versus Control

Among the 497 genes analyzed initially, fifteen genes, grey color, demonstrated significant differences in the fluorescence test compared with the control of FC > 1.1, out of which two genes, dark color, with FC > 1.5 were selected (Figure 1).

Table 1 illustrates statistically significant genes with FC > 1.5 and *P* value < 0.05.

### 3.3. Designation of Genes Differentiating Transcriptomes of HSC Specimens versus Control

Fifteen genes, dark color, demonstrated significant differences compared with the control of FC > 1.1, out of which 9 genes have FC > 1.5 and *P* < 0.05 (Figure 1). Three of the nine genes represent the expression of various isoforms (Table 2).

The largest increase in expression of genes was shown by MMP9 and TIMP. MMP9 expression is also increased in the HSC of the disease but this increase reaches a much lower level, which may indicate its importance both in the early stages of disease development and in progression. At the same time, in HSC specimens, isoforms of the ADAM17 gene expression were observed, which may be of particular interest in the mutual connection between the metalloproteinases and ADAMs.

In turn, the EphA2 gene expression in advanced stage colorectal cancer is at a lower level than in LSC tissues which can be explained by the increased importance of this gene in the initiation of tumor development.

## 4. Discussion

The above results are part of a series of ongoing researches into the molecular ground of carcinogenesis in colorectal cancer. The importance of matrix metalloproteinases, adamalizynes, EphA2, and other molecular factors, like TNF-*α*, is not fully understood. However, several observations point to their important role in the initiation and progression of cancer.

Existing data on the prognostic role of metalloproteinases and their inhibitors as markers of progression of colorectal cancer are ambiguous. Increased activity of MMP2 and MMP9 seems to play a key role in the growth and invasion of tumor and its metastasis [17]. MMP9 is particularly interesting, since a basic level of expression in most cells is generally low, whereas it is highly expressed in most human cancers and responds to growth factors and cytokines [18]. Collins et al. demonstrate a significant increase in the expression of MMP2 mRNA level in colon cancer cells with an increase of clinical stage (Duke C versus B), while the ratio of MMP2 to TIMP1 and TIMP2 did not change. The relationship between the expression of MMP2 and MMP9 mRNA and TIMP1 and TIMP2 also was not found. Zeng et al. estimated that the increase of MMP9 mRNA in colon cancer tissue relative to the level in healthy tissue of colorectal increased with progression of the disease and was negative predictor for disease-free survival and overall survival [19]. Other studies have shown that both protein levels of MMP2 and MMP9 were dependent on the severity scale Duke and the locating MMP9 assessed by immunohistochemistry were particularly active areas lying in the vicinity of the invasion of inflammatory cells.

The role of ADAM17 is complex, in particular, because of the direct relationship with the activity of TNF-*α*. ADAM17 is known as TNF-*α* converting enzyme (TACE); it is an essential factor for the appearance of the active form of TNF-*α*. This was confirmed in an animal model where the organism deprived of the ability to produce ADAM17 did not represent expression of active TNF-*α* [20]. A Polish study between 2004 and 2006 already demonstrated the role of TNF-*α* and its receptors TNFR2 and TNFR2/R7 (without exon 7) and the expression in colon cancer tissues, depending on clinical stage of disease. Molecular dysregulation of TNF system was observed in cancer tissue by an increased number of TNF-*α* receptors and ligand-receptor activity. It is interesting that expression of the receptors for TNF-*α* was highest in the metastatic cells (lymph nodes tissue) [21]. Furthermore, in subsequent studies, the largest number of mRNA copies for TNF-*α* and TNFR2/R7 in healthy cells surrounding cancer tissue in patients with stage III of colorectal cancer (compared to a group of lower degree of stage of the disease) was observed [22]. Probably, at the higher levels of clinical stage of the disease, molecular changes, which may have an effect in the progression of the disease, occur in the tissues surrounding the tumor. It is possible to draw a hypothetical line separating the anticancer activity of TNF-*α* and its influence on cancer progression and metastasis.

Observed in our study, overexpression of tumor necrosis factor (TNF) converting enzyme (TACE/ADAM17) in III and IV clinical stage CRC (from IIIst.) results, among others, activation of ADAM17-TNF-alpha axis, which has an impact on the progression of tumor growth, its invasiveness and ability to create distant metastases, which is reflected previously described overexpression of TNF-*α* in metastatic lymph nodes.

Probably the cellular level of ADAM17 may augment malignant potential of colon carcinoma cells by increasing their motility and expression of proangiogenic factors, while at the tissue level it enhances angiogenesis and affects the cross talk between tumor cells and immune system.

In turn, EphA2 overexpression in cancer cells is associated with decreased expression of E-cadherin which leads to loosening of the intercellular interaction [23]. The compound is studied, among others, by Herath and Boyd. They demonstrated that the tissues of colorectal cancer, an increase in the expression of EphA correlated with the coexistence of metastases to lymph nodes and liver [24]. In studies on therapeutic possibilities for patients with colon cancer with K-ras mutation, it was also found that the inhibition of EphA2 further reduces invasion and metastasis [25]. However, the authors found no other reports documenting increasing EphA2 gene expression primarily in the early stages of colorectal cancer.

Presented genes, especially ADAM17, MMP9, EphA2, TIMP1, ICAM 11, and CD4, may be used as prognostic markers of advanced stages of colorectal cancer, contributing to the development of new lines of therapy focused on reducing metastasis of the primary tumor.

For further exploring TACE/TNF-*α* pathway in CRC pathology, we plan to investigate the expression and localization of TACE in human colorectal cells and some of the effects of TNF-*α* release on migration process according to clinical and histopathological stage.

Moreover, in the present study, we focused on the analysis of genes that are overexpressed in colon cancer tissue. Significant path but also the analysis of genes that are silenced in the process of carcinogenesis and tumor progression. We wish that such research was a further step of our work. Further examination of the expression of genes associated with the migration process may allow for better knowledge and understanding of the processes occurring during the development of colon cancer. Such studies could help gene therapy allowing one to block tumor growth and its metastasis. Also, they could constitute the essence to identify new prognostic biomarkers in this type of cancer.

## Figures and Tables

**Table 1:** Genes differentiating control from a low stage of colorectal cancer.

ID	Gene	FC	Change (O)	*P* value
203499_at	EphA2	2.118391	O	0.03889112
203936_s_at	MMP9	2.2065341	O	0.004125773

O, overexpression.

**Table 2:** Genes differentiating colorectal cancer in the high stage of the control.

ID	Symbol	FC	Change (O)	*P* value
201666_at	TIMP1	3.7493954	O	1.3401924*E* − 8
202637_s_at	ICAM1	1.6801583	O	0.008369471
202638_s_at	ICAM1	1.6895063	O	0.02539259
203499_at	EPHA2	1.9644961	O	7.333963*E* − 4
203936_s_at	MMP9	4.0100565	O	8.0960683*E* − 7
205746_s_at	ADAM17	1.5147098	O	0.0093083875
212014_x_at	CD44	1.5726474	O	0.023357296
213532_at	ADAM17	1.5600195	O	0.0024534443
217523_at	CD44	1.7528782	O	0.0015765285

O, overexpression.

## References

[1] Wideł M. S., Wideł M. (2006). Mechanisms of metastasis and molecular markers of malignant tumor progression. I. Colorectal cancer. *Postȩpy Higieny i Medycyny Doświadczalnej*.

[2] Harlozińska-Szmyrka A., Sobańska E. (2008). Przerzuty nowotworowe—terrorysta XXI wieku. *Przewodnik Lekarza*.

[3] Popow-Woźniak A., Nowak D., Malicka-Błaszkiewicz M. (2009). Sposoby migracji komórek nowotworowych. *Postępy Biochemii*.

[4] Paduch R. (2005). Przerzut nowotworowy—znaczenie agregacji komórek nowotworowych z płytkami krwi. *Onkologia Polska*.

[5] Naduk-Kik J., Hrabec E. (2008). Udział metaloproteinaz macierzy w patogenezie cukrzycy i rozwoju retinopatii cukrzycowej. *Postępy Higieny i Medycyny Doświadczalnej*.

[6] Łukaszewicz-Zając M., Mroczko B., Szmitkowski M. (2009). Znaczenie metaloproteinaz oraz ich inhibitorów w raku żołądka. *Postępy Higieny i Medycyny Doświadczalnej*.

[7] Łukaszewicz M., Mroczko B., Szmitkowski M. (2008). Rola metaloproteinaz i ich inhibitorów w raku trzustki. *Postępy Higieny i Medycyny Doświadczalnej*.

[8] Kwiatkowski P., Godlewski J., Śliwińska-Jewsiewicka A., Kmieć Z. (2008). Cząsteczki adhezyjne w procesie nowotworzenia i przerzutowania. *Polish Annals of Medicine*.

[9] Śliwowska I., Kopczyński Z. (2005). Metaloproteinazy macierzy zewnątrzkomórkowej—charakterystyka biochemiczna i kliniczna wartość oznaczania u chorych na raka piersi. *Współczesna Onkologia*.

[10] Bogaczewicz J., Dudek W., Zubilewicz T. (2006). Rola metaloproteaz macierzy i ich tkankowych inhibitorów w angiogenezie. *Postępy Higieny i Medycyny Doświadczalnej*.

[11] Blanchot-Jossic F., Jarry A., Masson D. (2005). Up-regulated expression of ADAM17 in human colon carcinoma: co-expression with EGFR in neoplastic and endothelial cells. *Journal of Pathology*.

[12] Smalley D. M., Ley K. (2005). L-selectin: mechanisms and physiological significance of ectodomain cleavage. *Journal of Cellular and Molecular Medicine*.

[13] Hassemer E. L., Endres B., Toonen J. A., Ronchetti A., Dubielzig R., Sidjanin D. J. (2013). ADAM17 transactivates EGFR signaling during embryonic eyelid closure. *Investigative Ophthalmology and Visual Science*.

[14] Aydin D., Bilici A., Yavuzer D. (2015). Prognostic significance of ADAM17 expression in patients with gastric cancer who underwent curative gastrectomy. *Clinical and Translational Oncology*.

[15] McGowan P. M., McKiernan E., Bolster F. (2008). ADAM-17 predicts adverse outcome in patients with breast cancer. *Annals of Oncology*.

[16] Makrodouli E., Oikonomou E., Koc M. (2011). BRAF and RAS oncogenes regulate Rho GTPase pathways to mediate migration and invasion properties in human colon cancer cells: a comparative study. *Molecular Cancer*.

[17] Hsi-Hsien H., Wei-Syun H., Yueh-Min L. (2011). JNK suppression is essential for 17*β*-Estradiol inhibits prostaglandin E2-Induced uPA and MMP-9 expressions and cell migration in human LoVo colon cancer cells. *Journal of Biomedical Science*.

[18] Kang H., Jang S. W., Ko J. (2011). Human leucine zipper protein sLZIP induces migration and invasion of cervical cancer cells via expression of matrix metalloproteinase-9. *The Journal of Biological Chemistry*.

[19] Zeng Z.-S., Cohen A. M., Guillem J. G. (1999). Loss of basement membrane type IV collagen is associated with increased expression of metalloproteinases 2 and 9 (MMP-2 and MMP-9) during human colorectal tumorigenesis. *Carcinogenesis*.

[20] Black R. A., Rauch C. T., Kozlosky J. J. (1997). A metalloproteinase disintegrin that releases tumour necrosis factor-*α*. *Nature*.

[21] Duffy M. J., McKiernan E., O'Donovan N., McGowan P. M. (2009). Role of ADAMs in cancer formation and progression. *Clinical Cancer Research*.

[22] Muc-Wierzgon M., Nowakowska-Zajdel E., Kokot T. (2004). Genetic disregulation of gene coding tumor necrosis factor *α* receptors (TNF*α*Rs) in colorectal cancer cells. *Journal of Experimental & Clinical Cancer Research*.

[23] Muc-Wierzgon M., Nowakowska-Zajdel E., Kokot T. (2006). Genetic disregulation of TNF*α* type II receptors in colon cancer at the II and III stage of disease. *Journal of Biological Regulators and Homeostatic Agents*.

[24] Herath N. I., Boyd A. W. (2010). The role of Eph receptors and ephrin ligands in colorectal cancer. *International Journal of Cancer*.

[25] Saito T., Masuda N., Miyazaki T. (2004). Expression of EphA2 and E-cadherin in colorectal cancer: correlation with cancer metastasis. *Oncology Reports*.

